# Targeted protein delivery based on stimuli‐triggered nanomedicine

**DOI:** 10.1002/EXP.20230025

**Published:** 2023-11-23

**Authors:** Jinzhao Liu, Yang Zhou, Qingyang Lyu, Xiaotong Yao, Weiping Wang

**Affiliations:** ^1^ Department of Pharmacology and Pharmacy Li Ka Shing Faculty of Medicine The University of Hong Kong Hong Kong China; ^2^ State Key Laboratory of Pharmaceutical Biotechnology The University of Hong Kong Hong Kong China; ^3^ Dr. Li Dak‐Sum Research Centre The University of Hong Kong Hong Kong China; ^4^ Department of Chemistry Faculty of Science National University of Singapore Singapore Singapore

**Keywords:** protein delivery, protein‐based drugs, stimuli‐responsive nanoparticles, stimuli‐triggered targeting

## Abstract

Protein‐based drugs have shown unique advantages to treat various diseases in recent years. However, most protein therapeutics in clinical use are limited to extracellular targets with low delivery efficiency. To realize targeted protein delivery, a series of stimuli‐triggered nanoparticle formulations have been developed to improve delivery efficiency and reduce off‐target release. These smart nanoparticles are designed to release cargo proteins in response to either internal or external stimuli at pathological tissues. In this way, varieties of protein‐based drugs including antibodies, enzymes, and pro‐apoptotic proteins can be effectively delivered to desired sites for the treatment of cancer, inflammation, metabolic diseases, and so on with minimal side effects. In this review, recent advances in the design of stimuli‐triggered nanomedicine for targeted protein delivery in different biomedical applications will be discussed. A deeper understanding of these emerging strategies helps develop more efficient protein delivery systems for clinical use in the future.

## Graphical abstract

To realize targeted protein delivery, various stimuli‐triggered nanoparticle formulations have been developed. These smart nanoparticles are designed to release cargo proteins, such as antibodies, enzymes, and pro‐apoptotic proteins, in response to either internal or external stimuli. This review summarizes recent advances in the design of stimuli‐triggered nanomedicine for targeted protein delivery in different biomedical applications.

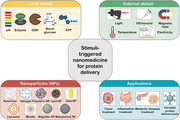

## 1 INTRODUCTION

Proteins are essential engines for cellular activities. Dysfunction of proteins can cause diseases, and protein‐based drugs are designed to modulate disordered functions and ameliorate pathological conditions.^[^ ^1^ ^]^ Protein‐based drugs such as insulin, enzymes and antibodies have demonstrated considerable potential in biological and medical applications.^[^ ^2^ ^]^ In 2022, the US Food and Drug Administration (FDA) approved 15 protein‐based drug candidates for the treatment of cancer, genetic disorder, metabolic diseases, etc., accounting for 40.5% of approved drugs.^[^ ^3^ ^]^ Compared with small drug molecules, protein‐based drugs have much higher specificity, efficacy and biocompatibility, demonstrating the superior characteristics of using proteins as therapeutics.^[^ ^4^ ^]^ Compared with gene therapeutics such as mRNA therapeutics, protein‐based drugs have inherent binding affinity to the targets, allowing for further drug conjugation such as antibody‐drug conjugate and antibody‐radionuclide conjugate.^[^ ^5^ ^]^ Protein‐based drugs can be incorporated with unnatural amino acid (UAA) to improve the bioactivity.^[^ ^6^ ^]^ Besides, certain protein therapeutics requires post translational modifications, which might not be dictated simply by mRNA sequences.^[^ ^7^ ^]^ However, the immunogenicity and susceptibility to degradation in blood circulation make it difficult for protein‐based drugs to reach and accumulate in the pathological tissues.^[^ ^8^ ^]^ Meanwhile, most of current protein‐based drugs are limited to the extracellular targets, due to their hydrophilicity, surface charge, and large molecular weight, which prevent endocytosis across cell membrane and successful endosome escape.^[^ ^9^ ^]^ As a result, there is an urgent need to explore effective protein delivery systems aiming to protect cargos from degradation and promote intracellular transportation.

Existing protein delivery strategies in FDA‐approved products include UAA incorporation and polyethylene glycol (PEG) coating.^[^ ^10^ ^]^ The former can extend blood circulation time and half‐life, but it is difficult to incorporate UAAs into proteins with large molecular weight in industrial production. The latter can reduce renal clearance and protein immunogenicity, but it is still hard for PEGylated proteins to be internalized into target cells. Nanomedicine refers to using nanoscale formulations for biomedical applications.^[^ ^11^ ^]^ To overcome these obstacles in protein delivery, a series of nanocarriers have been developed in the previous decades, such as liposomes, lipid nanoparticles, polymersomes, dendrimers, etc.^[^ ^12^ ^]^ They can encapsulate therapeutic proteins by either chemical conjugation or physical absorption. With the help of nanocarriers, proteins can be protected from enzymatic degradation during blood circulation and delivered into pathological tissues to exert biological functions.^[^ ^13^ ^]^ Although they are promising vehicles for protein delivery, a significant proportion of nanoparticles will be eliminated by mononuclear phagocyte system and accumulate in the liver, lung, and other healthy organs, leading to off‐target release and low delivery efficiency.^[^ ^14^ ^]^

**FIGURE 1:**
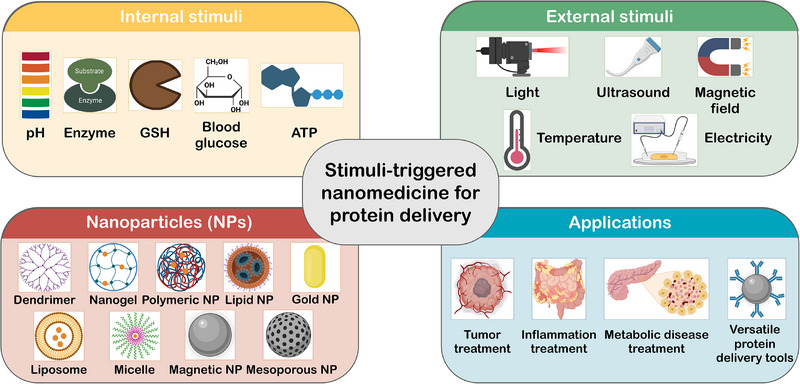
Schematic illustration of stimuli‐triggered nanomedicine for protein delivery. Protein‐based drugs can be encapsulated into a variety of nanocarriers with internal or external stimuli responsiveness to improve delivery efficiency for different biomedical applications.

To reduce off‐target delivery and unwanted side effects, stimuli‐triggered nanoparticle formulations have been explored for precise protein delivery.^[^ ^15^ ^]^ Upon specific stimulus, the structural change of nanoparticles will occur, leading to controlled protein release in target tissues or cells. Some of the stimuli‐triggered nanoparticles are responsive to internal stimuli, such as pH, enzymes, redox, glucose, etc., while the others are responsive to external stimuli, such as light, ultrasound, magnetic field, etc.^[^ ^16^ ^]^ By virtue of stimuli responsiveness, therapeutic proteins can be delivered to the target sites effectively to reduce adverse effects and achieve on‐demand dosing eventually. An applicable stimuli‐triggered nanomedicine for protein delivery should at least satisfy the following requirements. (i) The structures and functions of encapsulated proteins need to be preserved during the process of nanoparticle preparation. (ii) Nanoparticles should remain stable at physiological conditions with high serum tolerance. (iii) The encapsulated proteins can be released under specific stimuli. Herein, we will present an overview of recent stimuli‐triggered nanoparticle formulations for targeted protein delivery in different biomedical applications (Figure 1). The pros and cons of different strategies as well as future perspectives will also be discussed.

## 2 INTERNAL STIMULI RESPONSIVENESS

There are extensive differences in physicochemical characteristics between pathological and healthy tissues.^[^ ^17^ ^]^ Utilizing distinct features in the microenvironment of target sites, such as pH, enzymes, glutathione (GSH), etc., to trigger nanoparticle structural changes has been developed for precise protein delivery.^[^ ^18^ ^]^ In this way, after administration, protein delivery systems will automatically respond to the internal stimuli and release cargo proteins at target sites without any external intervention, which is quite convenient and effective in clinical use. In this part, different nanoplatforms with internal stimuli responsiveness for protein delivery will be introduced. The relevant studies in the review are summarized in Table 1.

**TABLE 1:** Representative examples of targeted protein delivery systems based on various stimuli‐responsive nanocarriers for different biomedical applications.

Stimuli	Responsive moieties	Nanocarriers	Cargo proteins	Applications	Ref.
pH	PEMA group	Polymersomes	Glucose oxidase	Lung cancer treatment	[22]
pH	*N*‐dibutylaminoethyl group	PAMAM dendrimers	RNase A	Cervical carcinoma treatment	[24]
pH	Catechol‐boronate ester group	PAMAM dendrimers	RNase A	Anti‐cancer treatment	[26a]
pH	Polyphenol	Polymeric nanoparticles	Anti‐TNF‐α antibody	Colitis treatment	[28]
Enzyme	GALGLP peptide	Nanogels	PON‐1	Atherosclerosis treatment	[31]
Enzyme	Hyaluronic acid	Nanogels	Cytochrome c	Lung cancer treatment	[33]
GSH	Disulfide bond	PAMAM dendrimers	RNase A, OVA, β‐galactosidase	Versatile protein carriers	[36]
GSH	Tannic acid (TA)	Mesoporous silica nanoparticles	Cytochrome c, β‐galactosidase	Versatile protein carriers	[38]
Blood glucose	4‐(Bromomethyl) phenylboronic acid	Lipid nanoparticles	Insulin	Diabetes treatment	[41]
ATP	Zn^2+^	ZIF‐90	RNase A, CRISPR/Cas9	Versatile protein carriers	[43]
MicroRNA	Single‐guide RNA	DNA nanoflowers	CRISPR/Cas9	Cervical carcinoma treatment	[44a]
ROS	Boronate group	Polypeptide nanoparticles	CRISPR/Cas9	Acetaminophen‐induced liver injury treatment	[44b]
Light	BODIPY group	PAMAM dendrimers	DNase I, OVA, Chymotrypsin	Versatile protein carriers	[48]
Light	DEACM group	PAMAM dendrimers	Glucose oxidase	Versatile protein carriers	[50b]
Light	Thioketal linker	HSA‐based nanoparticles	Anti‐CD47 antibody	Breast cancer treatment	[52]
Light	Gold nanorod	Gold nanorods	CRISPR/Cas9	Breast cancer treatment	[54]
Ultrasound	Perfluorocarbon	Perfluorocarbon nanoemulsions	Rabbit IgG	Versatile protein carriers	[57]
Ultrasound	HMME	ZIF‐8 nanoparticles	CRISPR/Cas9	Breast cancer treatment	[60]
Magnetic field	Fe_3_O_4_	Iron oxide nanoparticles	OVA	Cancer vaccine	[63]
Magnetic field	Iron oxide	Ferritin‐based nanoparticles	EGFP	Subcellular control of proteins and organelles	[64]
Temperature	DPPC	Exosome‐liposome hybrid nanoparticles	GM‐CSF	Anti‐cancer treatment	[65]
Electricity	Polypyrrole	Polypyrrole nanoparticles	Insulin	Versatile protein carriers	[67]

### 2.1 pH

Among internal stimuli, distinct pH values at target sites have been widely used to trigger protein release.^[^ ^19^ ^]^ For instance, tumor tissues, inflammatory tissues, and gastrointestinal (GI) tract have lower pH values compared with many other tissues. Within cells, lysosomes and endosomes also show acidic microenvironment.^[^ ^20^ ^]^ For nanocarriers, pH‐responsiveness mainly comes from acid‐cleavable chemical bonds or the protonation of ionizable groups. The former can cause changes of chemical structures, while the latter can cause the alterations of conformation or solubility, both of which can be employed to trigger cargo protein release under acidic condition.

**FIGURE 2:**
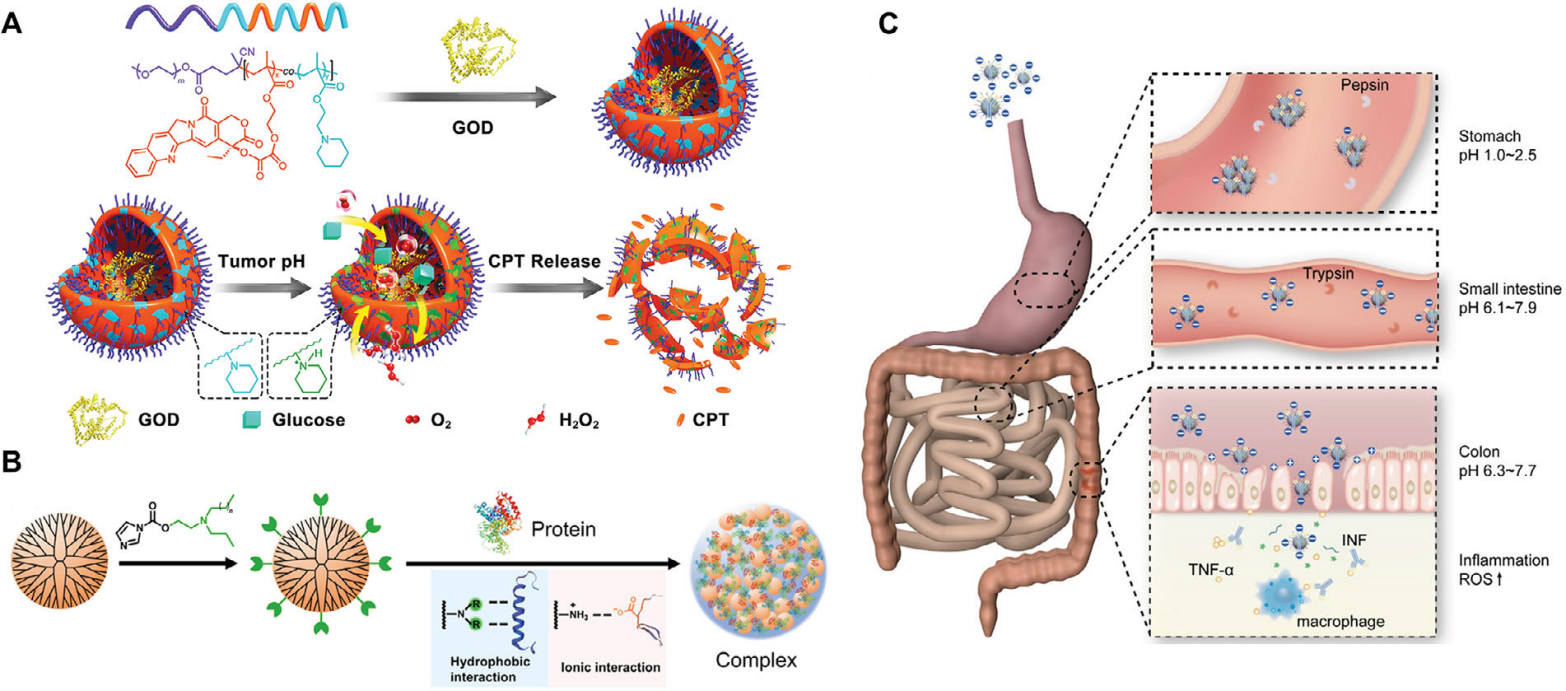
Designs of pH‐responsive nanoparticles for protein delivery. (A) The block copolymers composed of PEG and [P(CPTMA‐*co*‐PEMA)] can form polymersomes and load GOD into the interior aqueous chambers. The pH‐sensitive PEMA group is utilized to increase the permeability of the polymersome membranes, triggering hydrogen peroxide generation under the catalysis of GOD and subsequently releasing CPT from the polymeric prodrugs in lung cancer tissues. Reproduced with permission.^[^ ^22^ ^]^ Copyright 2017, American Chemical Society. (B) *N*‐dibutylaminoethyl‐modified PAMAM can encapsulate cargo proteins and facilitate endosomal escape. Reproduced with permission.^[^ ^24^ ^]^ Copyright 2021, Chinese Chemical Society. (C) Polyphenol nanoparticles can deliver anti‐TNF‐α antibodies to the inflamed colon tissues for the treatment of colitis via oral administration. Reproduced with permission.^[^ ^28^ ^]^ Copyright 2020, Ivyspring International Publisher.

Due to high glycolytic rate, tumor tissues generally have lower pH value in the range from 6.5 to 6.8.^[^ ^21^ ^]^ For anti‐cancer protein delivery, Li et al. developed a prodrug‐based polymersome nanoreactor to encapsulate glucose oxidase (GOD) for lung cancer treatment (Figure 2A).^[^ ^22^ ^]^ The block copolymers were composed of PEG and copolymerized monomers of camptothecin (CPT) and piperidine‐modified methacrylate [P(CPTMA‐co‐PEMA)], which could self‐assemble into polymersomes and load GOD within the interior aqueous chambers. After intravenous injection, polymersomes could accumulate at tumor tissues and the pH‐responsive PEMA segments were protonated in acidic tumor environment, thus causing high permeability of the polymersome membranes and free diffusion of glucose and oxygen into the nanoreactors. Under the catalysis of GOD, hydrogen peroxide would be produced to increase oxidative stress in tumor cells, and the oxalate linkage between CPT and polymer backbone would be cleaved under high concentration of hydrogen peroxide to release CPT for combinational therapy. Like PEMA, other pH‐responsive polymers including poly‐(α, β)‐DL‐aspartic acid (PASP), poly(β‐amino ester) (PAE) and poly(2‐(hexamethyleneimino)ethyl methacrylate) (PHMEMA) have also been reported for targeted protein delivery in response to acidic tumor environment.^[^ ^23^ ^]^

After endocytosis, proteases in the lysosome will catalyze protein hydrolysis under acidic microenvironment. Therefore, endosomal escape is necessary for intracellular protein delivery. Xu et al. designed an *N*‐dibutylaminoethyl modified polyamidoamine (PAMAM) dendrimer to encapsulate RNase A (Figure 2B).^[^ ^24^ ^]^ The hydrophobic *N*‐dibutylaminoethyl group could strengthen the binding affinity between PAMAM and proteins. Besides, it would become hydrophilic in acidic endosomes due to protonation effect. The protonation of tertiary amine could also destabilize endosome membrane, thereby facilitating endosomal escape of RNase A in HeLa cells. Similarly, 4‐diethylaminophenyl, diethylenediamine and magnesium phosphate have been developed as well for protein endosomal escape based on protonation effect.^[^ ^25^ ^]^ In addition, acid‐cleavable groups such as catechol‐boronate esters and Schiff‐bases could also promote pH‐triggered intracellular protein delivery.^[^ ^26^ ^]^

Oral protein delivery is a convenient administration route with high patient compliance, compared with intravenous or subcutaneous injection.^[^ ^27^ ^]^ However, challenges remain in the dynamic pH environment of GI tract. To address this issue, Wang et al. reported a polyphenol‐based nanoplatform to deliver anti‐TNF‐α antibodies for the treatment of colitis (Figure 2C).^[^ ^28^ ^]^ After oral administration, the nanoparticles first aggregated into large size (>300 nm) at acidic stomach environment because of the protonation to protect antibodies. The size could be reversed to 100 nm at neutral pH value after transport to the intestine. Finally, nanoparticles could penetrate the inflamed colon tissues with the help of smaller size and negative surface charge for efficient antibody delivery. Besides, Eudragit L100‐55 and sodium alginate have been reported as well for oral protein delivery as enteric coatings.^[^ ^29^ ^]^

### 2.2 Enzyme

Enzymes play a key role in biochemical reactions as biocatalysts. Altered enzyme expression levels are related to many diseases, which can be utilized as pathological signals to trigger protein release at target sites.^[^ ^30^ ^]^ For example, Basak et al. prepared PEG cross‐linked nanogels to encapsulate paraoxonase‐1 (PON‐1), an antioxidant protein to treat atherosclerosis.^[^ ^31^ ^]^ Atherosclerosis is characterized as pro‐inflammatory microenvironment with a high matrix metalloproteinase (MMP) expression level. After the nanogels reached pathological tissues, the GALGLP peptide sequence in the backbone of the nanogels would response to MMP in the microenvironment to initiate disintegration and protein release, which could inhibit the formation of macrophage foam cells and downregulate reactive oxygen species (ROS) level. Apart from GALGLP, other peptide sequences such as KPLELRAK, PVGLIG and PLGLAG have been reported as well for MMP‐responsive protein delivery.^[^ ^13^, ^32^ ^]^

In addition to MMP, Yang et al. designed a hyaluronidase (HAase)‐responsive nanogel formulation composed of PEGylated hyaluronic acid to load cytochrome c, an apoptotic protein, for tumor‐targeting delivery.^[^ ^33^ ^]^ The hyaluronic acid moieties could serve as active targeting ligands that bind to the surface of CD44‐overexpressing cancer cells and promote nanoparticle accumulation in tumor tissues.^[^ ^34^ ^]^ After the nanoparticles entered cancer cells, upregulated HAase level in the cytosol would initiate the degradation of nanogel backbone, thereby releasing cytochrome c to effectively induce cancer cell apoptosis.

### 2.3 GSH

GSH is a tripeptide that controls redox homeostasis inside cells and is responsible for the reductive microenvironment at some lesion sites like tumor tissues.^[^ ^35^ ^]^ Variation of GSH content has been explored as an endogenous trigger for GSH‐responsive protein delivery. For instance, Zhang et al. designed a cross‐linked polymer‐protein nanoparticle formulation for intracellular protein delivery.^[^ ^36^ ^]^ The polymer used was PAMAM dendrimer grafted with phenylboronic acid, which was cross‐linked with cargo proteins through di(4‐nitrophenyl)−2,2′‐dithiodiethanocarbonate (DND) via mild reactions. The cross‐linked nanoparticles exhibited high serum stability and effective cell internalization. After endocytosis, disulfide bond in the linker would be cleaved attributed to higher intracellular GSH concentration in cancer cells, allowing the cytosolic release of native cargo proteins. In addition to being a cross‐linker, disulfide bond has also been incorporated into the core of PAMAM or silica nanocapsules for GSH‐triggered protein release.^[^ ^37^ ^]^

Apart from disulfide bond, the competitive non‐covalent interactions can also be employed for GSH‐responsiveness. Han et al. developed a polyphenol‐based nanoplatform for intracellular protein delivery.^[^ ^38^ ^]^ They chose mesoporous silica nanoparticles as the core of the nanoplatform, with cytochrome c self‐assembled with tannic acid (TA) on the surface. After cellular uptake, the higher GSH content in the cytosol of cancer cells could initiate nanoparticle disassociation due to the competing supramolecular interactions between GSH with both TA and cargo proteins, thereby releasing cytochrome c to promote cell apoptosis.

### 2.4 Other internal stimuli

**FIGURE 3:**
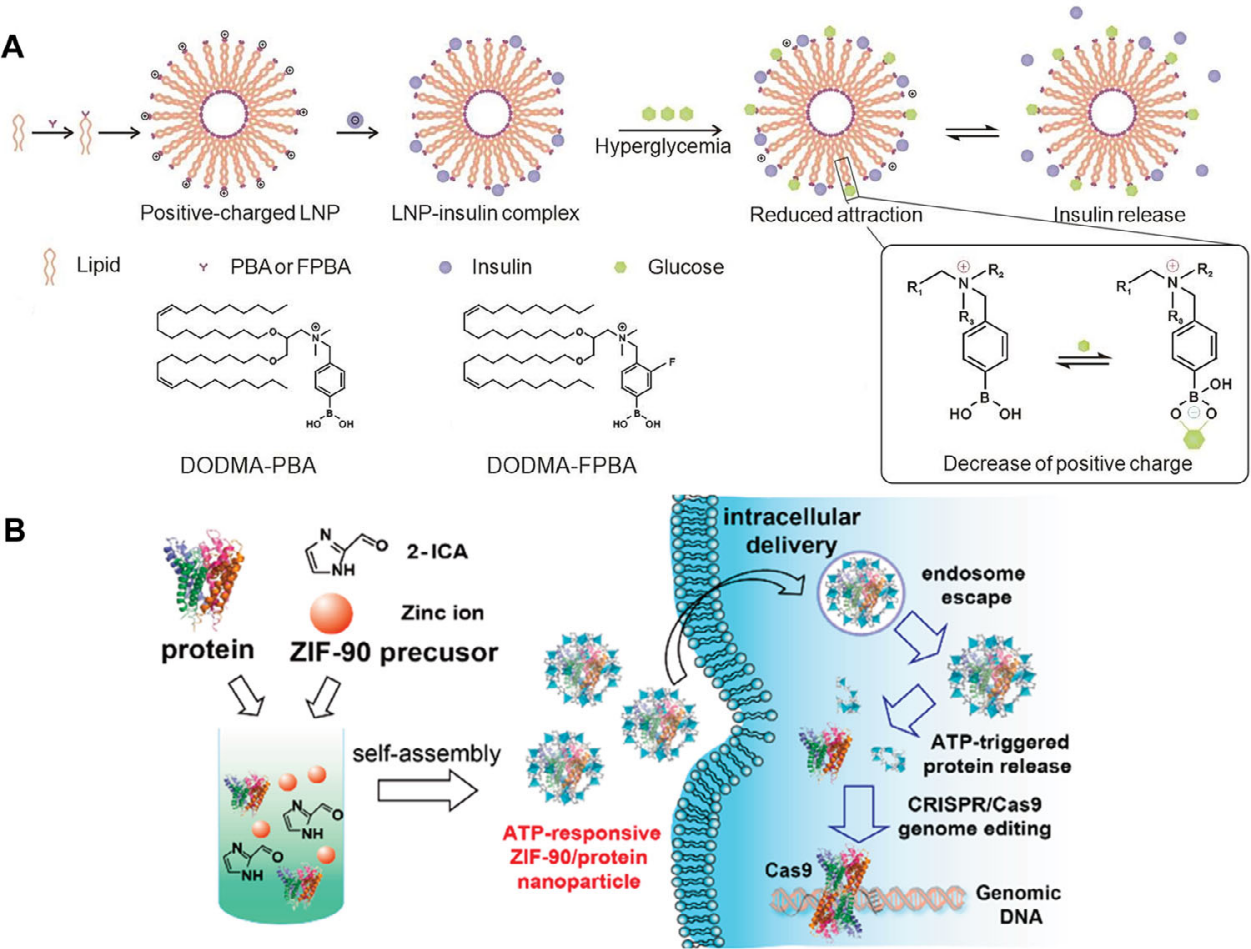
Strategies of glucose or ATP‐responsive protein delivery. (A) PBA‐modified DODMA can help load insulin via electrostatic interactions and trigger insulin release under hyperglycemia environment. Reproduced with permission.^[^ ^41^ ^]^ Copyright 2023, Wiley‐VCH. (B) ZIF‐90 nanoparticles can encapsulate protein‐based drugs and release them in the cytosol at high ATP concentrations. Reproduced with permission.^[^ ^43^ ^]^ Copyright 2019, American Chemical Society.

Blood glucose (BG) is the major energy source for cells. Pancreatic β cells can secrete insulin to maintain BG balance and reduce hyper‐ and hypoglycemia risk.^[^ ^39^ ^]^ Type 1 diabetes patients need multiple subcutaneous injections of insulin to mimic endogenous insulin secretion and control the BG level. However, current insulin formulations cannot react to BG fluctuation and have a risk of life‐threatening hypoglycemia.^[^ ^40^ ^]^ To solve this problem, Liu et al. developed a glucose‐responsive lipid nanoparticle formulation to deliver insulin (Figure 3A).^[^ ^41^ ^]^ The lipid nanoparticles were composed of 4‐(Bromomethyl)phenylboronic acid (PBA)‐modified 1,2‐dioleyloxy‐3‐dimethylamino‐propane (DODMA), and the positively charged surface could load negatively charged insulin by electrostatic attraction. Under hyperglycemia environment, PBA groups would bind to glucose and bring negative charges to the nanoparticles to reduce electrostatic interaction, eventually leading to glucose‐triggered insulin release. After subcutaneous injection, nanoparticles could extend the normoglycemia duration, and regulate BG level timely after intraperitoneal glucose administration in the diabetic mice. Aside from DODMA, PBA moieties could also be conjugated to hyaluronic acid to achieve glucose‐responsive oral insulin delivery.^[^ ^42^ ^]^

Compared with BG, adenosine triphosphate (ATP) is the energy‐carrying molecule that cells can directly use. Due to the abnormal glycolysis in tumor tissues, their ATP concentration is higher than healthy tissues, which can be utilized as the internal stimulus to trigger protein release. As a proof‐of‐concept, Yang et al. reported an ATP‐responsive protein delivery system based on zeolitic imidazole framework‐90 (ZIF‐90) (Figure 3B).^[^ ^43^ ^]^ The nanoparticles were made of imidazole‐2‐carboxaldehyde and Zn^2+^, and proteins were loaded into the core of nanocrystalline shells via biomimetic mineralization with a high encapsulation efficiency above 90%. Owing to the competitive coordination between Zn^2+^ and ATP, ZIF‐90 nanoparticles could be disassociated in HeLa cells at a high intracellular ATP concentration, thus causing the release of preloaded RNase A into the cytosol. Besides the internal stimuli mentioned in this part, the others including microRNA and ROS have also been employed for precise protein delivery in response to inherent pathological microenvironments.^[^ ^44^ ^]^

## 3 EXTERNAL STIMULI RESPONSIVENESS

In comparison to internal stimuli, externally applied stimuli exhibit better controllability for targeted protein delivery. External stimuli‐responsive delivery systems are designed to trigger cargo release with high spatio‐temporal resolution in response to light, ultrasound, magnetic field etc. The parameters of external stimuli such as location, intensity, and duration can be easily adjusted to improve therapeutic efficacy and reduce off‐target release of proteins. In this part, we will discuss recent advances of external stimuli‐responsive nanomedicines used for protein delivery.

### 3.1 Light

As an attractive non‐invasive source, light can be applied to control drug release at desired sites with high spatio‐temporal precision.^[^ ^45^ ^]^ To fabricate photoresponsive nanomaterials, photoremovable protecting groups (PPGs), photosensitizers and photothermal agents can be utilized to trigger photocleavage, produce ROS and generate heat, respectively, upon light irradiation. Regarding the wavelength, near‐infrared (NIR) range (650–900 nm) with deep tissue penetration and low phototoxicity is preferred in therapeutic applications.^[^ ^46^ ^]^ For the skin and eye, in addition to NIR, visible light is also applicable to penetrate surface tissues and reach the lesions.^[^ ^47^ ^]^

**FIGURE 4:**
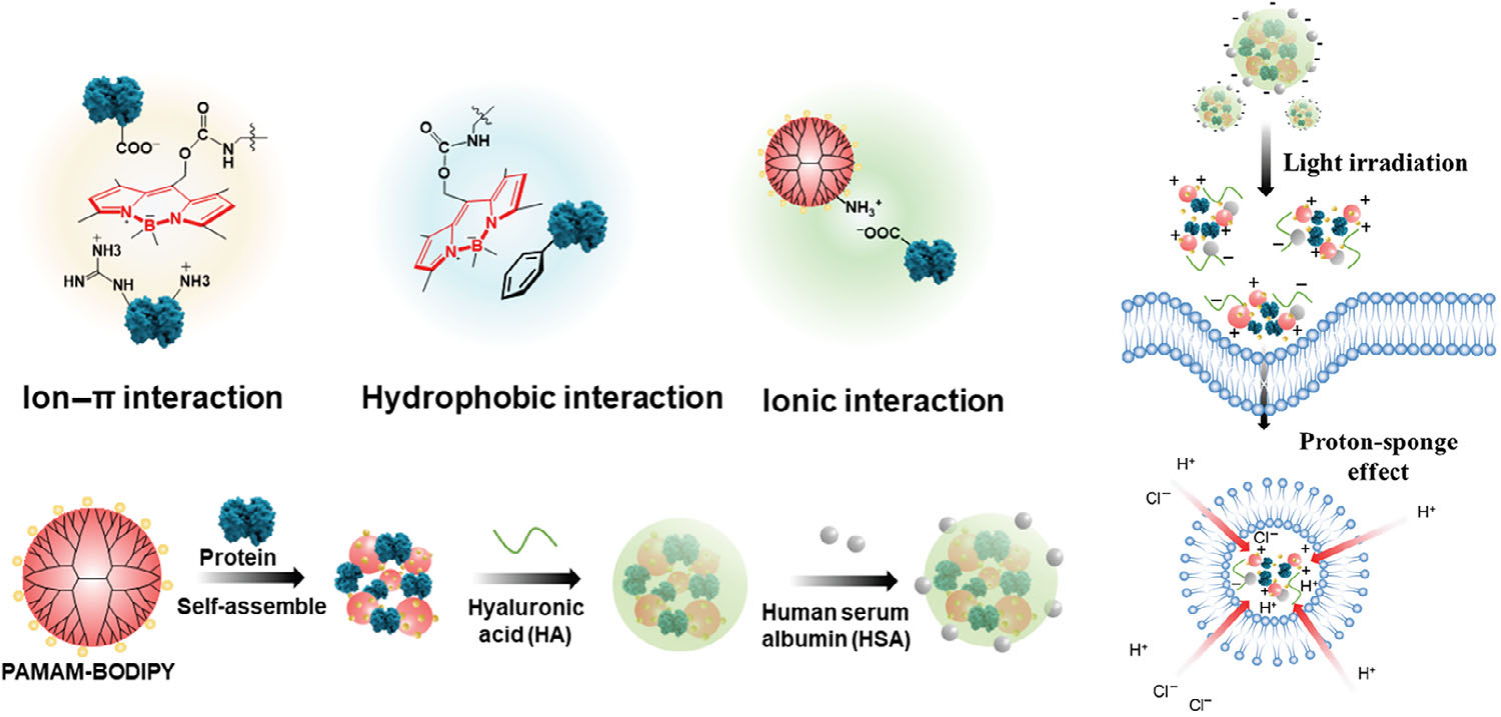
Light‐enhanced cytosolic protein delivery system. BODIPY‐modified PAMAM can form nanocomplexes with proteins via hydrophobic and electrostatic interactions. Nanoparticles under light irradiation can promote intracellular delivery of multiple cargo proteins and facilitate endosomal escape. Reproduced with permission.^[^ ^48^ ^]^ Copyright 2022, Tsinghua University Press.

To achieve light‐enhanced cytosolic protein delivery, Zhou et al. designed a boron‐dipyrromethene (BODIPY) modified PAMAM nanoplatform (Figure 4).^[^ ^48^ ^]^ BODIPY was a green light‐responsive PPG with suitable photosensitivity. After conjugation, the aromatic rings of BODIPY PPGs could interact with the guanidinium groups and carboxylate groups of protein residues via ion‐π interactions. In addition, hydrophobic BODIPY PPGs could also interact with hydrophobic residues of proteins. Both types of interactions could help improve the protein encapsulation efficiency. Further coatings with hyaluronic acid and human serum albumin (HSA) endowed nanoparticles with negative charge and increased serum stability. Upon light irradiation (520 nm, 25 mW cm^−2^, 5 min), BODIPY moieties would be cleaved and nanoparticles were dissociated into positively charged fragments, leading to the cytosolic delivery and endosomal escape of cargo proteins. Moreover, the nanoplatform can be modified to be NIR‐light responsive through modification of BODIPY moieties, allowing incident light to penetrate deeply into body tissues.^[^ ^49^ ^]^ Apart from BODIPY, nitroveratryloxycarbonyl (NVOC) and 7‐diethylamino‐4‐hydroxymethylcoumarin (DEACM) groups were reported as well for UV/blue light‐triggered protein delivery.^[^ ^50^ ^]^ To realize long‐wavelength excitation, upconversion nanoparticles were designed to convert NIR light (980 nm) into UV light to cleave nitrobenzyl groups and release caged proteins.^[^ ^51^ ^]^

Besides photocleavage, photodynamic therapy (PDT) that requires photosensitizers to absorb photon energy and generate ROS can also be used for photoresponsive protein delivery. For instance, Lu et al. developed an HSA‐based nanoplatform for CD47‐blocking immunotherapy.^[^ ^52^ ^]^ Anti‐CD47 antibodies were cross‐linked with HSA through ROS‐responsive thioketal linkers to form nanoparticles. Photosensitizer IR820 was linked to the amino groups of proteins via *N*‐hydroxysuccinimide conjugation. Nanoparticles were further coated with PEG to improve their stability. After intravenous injection, upon NIR light irradiation at tumor sites (808 nm, 1 W cm^−2^, 5 min), IR820 could produce ROS and cleave thioketal linkers to release antibodies in the tumor microenvironment. Besides thioketal linker, the other ROS‐responsive moieties such as unsaturated lipids and phenylboronic acid have been explored as well for light‐responsive protein delivery based on PDT. ^[^ ^53^ ^]^

Another photoresponsive strategy is photothermal therapy (PTT), which utilizes photothermal agents to transfer NIR photon energy into heat. For example, Huang et al. fabricated a cancer cell membrane‐derived nanocarrier to deliver clustered regularly interspaced short palindromic repeat (CRISPR)/CRISPR‐associated protein 9 (Cas9).^[^ ^54^ ^]^ The positively charged Cas9 proteins were encapsulated by electrostatic interactions with MDA‐MB‐231 cell membrane. Single‐guide RNAs targeting survivin gene were loaded onto the gold nanorods, which were further extruded with Cas9‐loaded membrane to obtain the photoresponsive nanoformulation. After intravenous injection, nanoparticles could accumulate in tumor tissues due to homologous targeting effect of the outer membrane. Upon NIR light irradiation (808 nm, 1 W cm^−2^, 5 min), gold nanorods were served as photothermal agents to cause hyperthermia, which could destroy the structure of coated membrane and promote ribonucleoprotein release for gene therapy. Aside from gold nanorods, polydopamine was also employed as PTT agents for photoresponsive GOD delivery.^[^ ^55^ ^]^

### 3.2 Ultrasound

Ultrasound is widely used in biomedical applications. Low‐power ultrasound can be employed for diagnosis, while the high‐power one can be used to remove pathological tissues.^[^ ^56^ ^]^ As a safe external stimulus, ultrasound has been utilized to trigger protein release with spatio‐temporal control and high tissue penetration depth. For instance, Sloand et al. designed a perfluorocarbon nanoplatform for ultrasound‐sensitive cytosolic protein delivery.^[^ ^57^ ^]^ The cargo proteins such as rabbit IgG were complexed non‐covalently with fluoro‐amphiphilic molecules to ensure the efficient dispersion within fluorous nanocarriers via fluorine‐fluorine interactions. The nanoparticles were further coated with fluorinated RGD peptide for active targeting effect. Upon localized to A549 cell surface, perfluorocarbon nanoparticles would undergo liquid–gas phase transition and produce microbubbles in response to the ultrasound (1 MHz, 2 W cm^−2^, 72 s), leading to the formation of transient pores on cell membrane. Rabbit IgG could be simultaneously delivered into the cytoplasm with high efficiency. Similarly, ultrasound‐triggered cavitation has also been explored to perturb blood vessel wall or cell membrane with SF_6_ microbubbles for protein delivery.^[^ ^58^ ^]^

**FIGURE 5:**
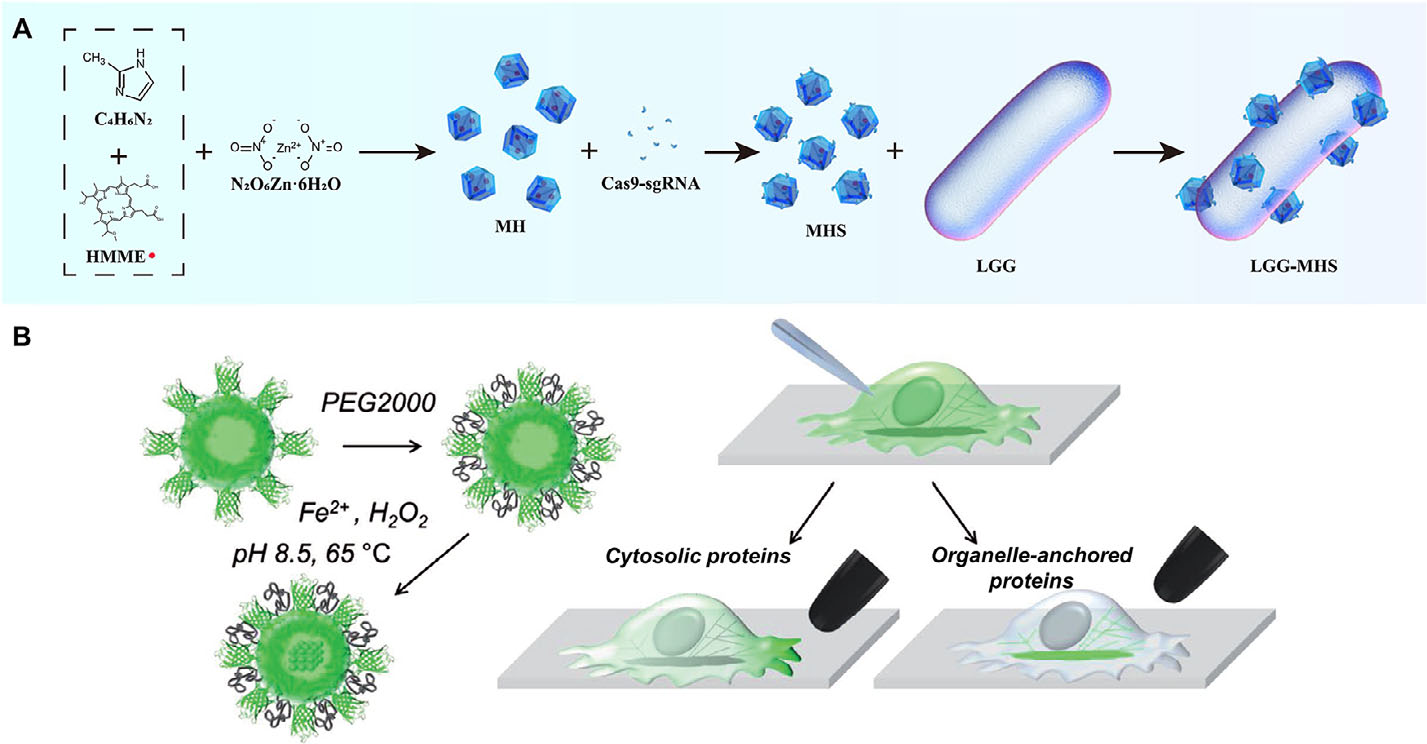
Designs of ultrasound or magnetic field‐responsive protein delivery systems. (A) ZIF‐8 nanoparticles can encapsulate sonosensitizer HMME and CRISPR/Cas9. Under ultrasound application, this system will produce ROS and release CRISPR/Cas9 in the cytoplasm for gene therapy. Reproduced with permission.^[^ ^60^ ^]^ Copyright 2022, Springer Nature. (B) Recombinant protein nanoparticles are loaded with magnetic core and grafted with PEG chains. After microinjection, cytosolic proteins or organelle‐anchored proteins that are fused to anti‐EGFP antibodies will be rapidly targeted by nanoparticles. The magnetic tip can help either control the localization of cytosolic proteins or manipulate organelle functions. Reproduced with permission.^[^ ^64^ ^]^ Copyright 2017, Wiley‐VCH.

The other ultrasound‐controlled strategy is through sonodynamic therapy (SDT). Like PDT, it requires sonosensitizers to generate ROS upon specific ultrasound triggering.^[^ ^59^ ^]^ The major difference between PDT and SDT is their energy sources. PDT utilizes light, while SDT employs ultrasound, which can reach deep‐seated disease sites with ease. For example, Yu et al. reported a probiotic‐ZIF‐8 nanosystem to deliver CRISPR/Cas9 targeting indoleamine 2,3‐dioxygenase‐1 (IDO1) (Figure 5A).^[^ ^60^ ^]^ ZIF‐8 nanoparticles were made of Zn^2+^ and 2‐methylimidazole, with sonosensitizer hematoporphyrin monomethyl ether (HMME) encapsulated in the core and CRISPR/Cas9 absorbed on the surface. Afterwards, nanoparticles were compounded with probiotics *Lactobacillus rhamnosus GG* (LGG) via electrostatic interactions. After intravenous injection, nanocomplexes could accumulate at tumor tissues due to the hypoxia‐targeting capacity of LGG. Under ultrasound application (1 MHz, 1 W cm^−2^, 150 s), HMME could generate ROS to initiate endosomal/lysosomal rupture and release cargo proteins in the cytoplasm for combinational immunotherapy. Similar SDT designs were also applied to liposomes or other metal‐organic frameworks for ultrasound‐controlled protein delivery.^[^ ^61^ ^]^

### 3.3 Magnetic field

Magnetic field is another non‐invasive exogenous stimulus and plays an important role in diagnosis and treatment. Magnetically controlled strategies rely on an extracorporeal magnetic field and magnetic metal‐based materials, which can effectively promote protein accumulation at desired sites.^[^ ^62^ ^]^ For instance, Huang et al. developed an Fe_3_O_4_‐based nanovaccine for cytoplasmic delivery of tumor antigens.^[^ ^63^ ^]^ Fe_3_O_4_ could self‐assemble with ovalbumin (OVA), a model antigen, to form magnetic nanoparticles, which were further coated with CaCO_3_ and MnCO_3_ to alleviate the aggregation. After subcutaneous injection, nanoparticles could actively deliver OVA to the cytoplasm of dendritic cells with the help of magnetic field. The released OVA could promote dendritic cell maturation and antigen presentation to activate CD8^+^ T cells and memory T cells. At the same time, intracellular Mn^2+^ and Ca^2+^ levels were increased after the degradation of outer layer, which could serve as immunologic adjuvants to enhance the immune response of OVA.

Apart from intracellular protein delivery, magnetic field can also be employed to control protein activity in subcellular level. As a proof‐of‐concept, Liße et al. designed a ferritin‐based nanoplatform for magnetic manipulation of proteins inside living cells (Figure 5B).^[^ ^64^ ^]^ They firstly prepared a recombinant protein composed of enhanced green fluorescent protein (EGFP) and human ferritin. The recombinant protein could self‐assemble into nanoparticles, which were further loaded with a magnetite core and grafted with PEG chains. To equip target protein Tom20 in the mitochondria with magnetically controllable properties, Tom20 was fused to anti‐EGFP antibody so that the EGFP moieties of nanoplatform could recognize Tom20. After microinjection of nanoparticles into HeLa cells, PEG layer would protect them from fast degradation and ensure free diffusion so that the nanoparticles could bind with Tom20 adequately. Upon applying magnetic tip close to cell membrane, Tom20 would move towards the tip, thus causing the arrest of mitochondrial dynamics. This work demonstrated the potential of magnetic nanomaterials for subcellular control of proteins and organelles.

### 3.4 Other external stimuli

Temperature is also one of the external stimuli for controlled protein delivery systems. Apart from photothermal therapy using light to generate heat mentioned in Section 3.1, the other method is hyperthermic intraperitoneal chemotherapy (HIPEC), which involves intraperitoneal drug filling with heating and has already been used for metastatic peritoneal carcinoma treatment. To further promote drug penetration and reverse immunosuppression microenvironment, Lv et al. designed a thermosensitive exosome‐liposome hybrid nanoparticle formulation.^[^ ^65^ ^]^ The fibroblasts were firstly transfected for overexpression of CD47 and GM‐CSF. Then, the exosomes of fibroblasts were collected by ultracentrifugation with CD47 on the membrane and GM‐CSF inside. Finally, the engineered exosomes were fused with thermo‐sensitive liposomes. After intravenous administration, CD47 on the membrane could help to escape from clearance by the mononuclear phagocytic system and nanoparticles could accumulate into tumor tissues. The GM‐CSF protein would be released at the hyperthermia condition of HIPEC, which could promote the repolarization of macrophages to M1 phenotype to activate anti‐cancer immunity and inhibit cancer metastasis.

Another external stimulus for protein delivery is electricity. Compared with other exogenous stimuli, complex instrument is not required to generate electric signals, and electronic devices can be developed to wireless implants in the future.^[^ ^66^ ^]^ For instance, Hosseini‐Nassab et al. developed a polypyrrole nanoparticle formulation to deliver insulin.^[^ ^67^ ^]^ Electrically conducting polypyrrole nanoparticles were prepared by microemulsion with insulin absorbed on the surface via electrostatic and hydrophobic interactions. Upon electrical stimulation (−1 V vs Ag/AgCl), insulin would be released under in vitro condition due to the altered strength of interactions between protein and conducting polymers. Moreover, the released insulin still remained bioactivity with hypoglycemic effect in vivo. Similarly, electrical stimulation was also applied for controlled insulin release based on reduced graphene oxide interface and gold‐polypyrrole nanocomposite.^[^ ^68^ ^]^

## 4 SUMMARY AND PERSPECTIVE

Nanoparticle formulation is one of the most promising approaches for targeted protein delivery. To reduce off‐target release and improve therapeutic efficacy of proteins, various stimuli‐triggered nanoparticles have been developed in the past decade. In this way, a series of protein therapeutics such as insulin, antibodies, enzymes, and pro‐apoptotic proteins can be encapsulated into smart nanocarriers with endogenous or exogenous stimuli responsiveness for the treatment of cancer, inflammation, metabolic diseases, etc. For internal stimuli responsiveness, nanoparticles are designed to respond to a specific stimulus at pathological tissues and release cargo proteins without any external intervention, which is quite convenient and effective in practice. However, it is difficult to precisely control these internal triggers due to the variations from individuals, such as pH values at tumor sites and MMP expression levels in inflammatory tissues.^[^ ^69^ ^]^ From this point of view, genetic testing of pathological tissues might be helpful in the future to accurately predict disease phenotypes and determine internal responsiveness.^[^ ^70^ ^]^

For external stimuli responsiveness, nanoparticle delivery systems can achieve targeted protein delivery with high controllability and unprecedented spatio‐temporal resolution. Protein release profiles can be easily manipulated through the adjustment of stimulus parameters including location, intensity, and duration period. However, limited tissue penetration depth and imprecise focusing might be current issues of external stimuli.^[^ ^71^ ^]^ Therefore, developing long‐wavelength photoresponsive materials and high‐sensitive ultrasound‐controlled systems is preferred for the treatment of deep‐seated diseases.^[^ ^72^ ^]^ Theragnostic materials and imaging agents are useful to determine the focus location of stimulus and avoid adverse effects to normal tissues.^[^ ^73^ ^]^ Based on these strategies, internal or external stimuli‐triggered nanomedicine still hold considerable potential for targeted protein delivery in clinical use.

The vast majority of FDA‐approved protein‐based therapeutics are monoclonal antibodies, bispecific antibodies, and antibody‐drug conjugates.^[^ ^74^ ^]^ However, most of them are restricted to the extracellular targets such as PD‐1/PD‐L1, CTLA‐4, and CD3, due to the hydrophilicity, surface charge, and large molecule weight.^[^ ^3^ ^]^ For cytosolic protein delivery, various stimuli‐triggered nanoplatforms have been developed to prevent enzymatic degradation during circulation, promote endocytosis across cell membranes, and facilitate endosomal escape inside cells. However, there are still many problems to be addressed for their translation from the bench to the bedside. For instance, some of them have sophisticated structures in response to different stimuli, which makes large‐scale production quite complex. Besides, the bioactivities of protein‐based drugs need to be carefully preserved during nanoparticle fabrication, which requires a suitable manufacturing process and strict quality control.^[^ ^75^ ^]^ Thirdly, the optimization of internal stimuli from animal models to patients as well as the clinical settings of external stimuli need to be explored in the future to promote clinical translation. In addition, it is crucial to define the safe dosage of stimuli‐triggered nanomedicine and the strength of applied stimulus before clinical trials. Detailed information on stimuli‐responsiveness, systemic immune response and genotoxicity should be investigated thoroughly in pre‐clinical animal models to avoid potential side effects in the patients.^[^ ^62b^ ^]^ Finally, the large‐scale production cost of stimuli‐triggered protein delivery systems and the cost of medical devices to generate respective external stimulus also need to be considered for broader clinical applications.

Protein‐based therapeutics have demonstrated superior potential in biomedical applications. Most of FDA‐approved protein drugs require intravenous or subcutaneous injection to enter blood circulation, interact with target molecules and exert biological functions.^[^ ^76^ ^]^ To improve patient compliance, oral protein delivery systems are one of the promising therapeutic strategies with high convenience. Oral administration needs suitable carriers to protect proteins in the GI tract and go through the epithelial layer of intestine. From this view, nanoparticle formulation with enteric coatings will be a good choice in the future to achieve oral protein delivery. The targets of some protein drugs are located in the organelles, such as mitochondria and nucleus.^[^ ^77^ ^]^ Therefore, organelle‐targeting nanoplatforms with specific affinity ligands or stimuli responsiveness will be useful to achieve subcellular delivery and improve therapeutic efficacy of the delivered proteins. With rapid development of nanoparticle formulations and stimuli‐responsive materials, it is likely that stimuli‐triggered nanomedicine will be widely used for targeted protein delivery in clinical applications and realize precision medicine eventually.

## CONFLICT OF INTEREST STATEMENT

The authors declare no conflicts of interest.
